# Co-designing behavioural activation for depression for autistic adolescents: A case series

**DOI:** 10.1177/13591045241229583

**Published:** 2024-01-29

**Authors:** Zameer Mohamed, Ailsa Russell, Melanie Palmer, Emily Simonoff, Matthew J Hollocks

**Affiliations:** 1 Institute of Psychiatry, Psychology and Neuroscience, 4616 King’s College London, London, UK; 2 Centre for Applied Autism Research, Department of Psychology, 1555 University of Bath, Bath, UK; 3 South London and Maudsley NHS Trust, London, UK

**Keywords:** Autism, depression, treatment, co-design, acceptability

## Abstract

Autistic youth are at high risk of depression**,** but there are few psychological interventions that have been specifically designed for use with this population. Behavioural activation (BA) is a particularly promising approach for autistic adolescents, having an established evidence-base for the treatment of depression in non-autistic people, and with a strong focus on behavioural, rather than cognitive change, which is a challenge for some autistic people. In this study, we worked with autistic adolescents and clinicians to co-design a BA-informed intervention to be delivered in an online format. We then conducted a pilot case-series with seven autistic adolescents with depression. Our focus was on establishing the acceptability and feasibility of the intervention but clinical outcomes on both self- and parent-reported symptoms of depression and anxiety are also presented. Our results indicate the intervention to be acceptable and feasible for autistic adolescents, with six out of seven participants being retained to the end of the intervention. Qualitative feedback indicated that all participants found the intervention a positive experience and would recommend it to others. Similarly, all participants found the online format acceptable, with 64% preferring this format to face-to-face therapy. Qualitative feedback and suggestions for refinement will also be discussed.

## Plain language summary

Autistic youth are at high risk of depression, but there are few psychological interventions that have been specifically designed for use with this population. Behavioural activation (BA) is a particularly promising approach for autistic adolescents, which has been used previously with non-autistic people. BA-focusses on improving mood through increasing engagement in positive activities and is well suited to being adapted to meet the needs of autistic youth. In this study, we worked with autistic adolescents and clinicians to co-design a BA-informed intervention to be delivered in an online format. We then conducted a pilot case-series with seven autistic adolescents with depression. Our focus was on establishing the acceptability (can participants complete the intervention) and feasibility (can this be done again on a larger scale) of the intervention. Our results indicated that the intervention was acceptable and feasible for autistic adolescents, with six out of seven participants being retained to the end of the intervention. Feedback from young people and their parents indicated that all participants both found the intervention a positive experience and would recommend it to others. Similarly, all participants found the online format acceptable, with 64% preferring this format to face-to-face therapy. Qualitative feedback and suggestions for refinement will also be discussed.

Autistic youth are at high risk of experiencing major depression, with a point prevalence rate of around 10% in adolescence (Hollocks et al., 2022). This is twice the expected rate compared to those without an autism diagnosis, where the prevalence estimate is around 4%–5% (Costello et al., 2005). Addressing depression in the adolescent period is of major importance with estimated rates doubling to around 20% by adulthood (Hollocks et al., 2019). Depression in autistic individuals has been associated with a range of difficulties, including lower quality of life (Lawson et al., 2020), increased suicidality (Cassidy et al., 2018), and an increased impact on caregivers (Cadman et al., 2012). Despite this recognition of both the high prevalence and impact of depression for autistic youth, to date, little progress has been made on the development of effective psychosocial interventions.

Evidence from studies investigating anxiety treatments for autistic individuals have demonstrated that adapting psychological interventions, such as cognitive behavioural therapy (CBT) can lead to effective interventions (Wood et al., 2020). There has been limited research with regards to psychosocial treatments for depression in autism when compared to anxiety (White et al., 2018). The evidence that does exist tends to be based on adult populations and suggests that CBT approaches are effective, but with effect sizes in the small to moderate range (Witchers et al., 2022). Many of the more effective studies draw heavily on behavioural elements (Menezes et al., 2020), such as behavioural activation (BA) which may make this a key ingredient in any successful intervention, having reduced cognitive and language demands when compared to techniques such as reappraisal.

BA is a well-established intervention approach for depression in non-autistic adults (Ekhers et al., 2014), showing comparable outcomes compared to CBT (Richards et al., 2016), and has more recently been used with adolescents (Pass et al., 2018). BA is based on the contextual model of depression (Jacobson et al., 2001) and places an emphasis on promoting a positive mood through increased participation in activities that are meaningful to the individual (Santos et al., 2019). This predicts that reductions in engagement with activities that generate positive reinforcement results in a reduction is positive affect, lower motivation and ultimately in a depressed mood (Carvalho & Hopko., 2011). Recently, Russell et al. (2020) have adapted BA for use with autistic adults in the form of a low intensity psychological intervention. Low intensity refers to the provision of evidence-based information which individuals are supported to personalise through meetings with a therapist or coach. An initial feasibility and acceptability study found that the low intensity guided self-help intervention was feasible and acceptable to participants (Horwood et al., 2021). However, such a low intensity approach may not be well suited to adolescent depression, particularly with autistic young people who likely need enhanced support from therapists and some degree of parent/carer involvement due to difficulties in areas such as planning/executive functioning (Demetriou et al., 2018).

In addition to the development of adapted evidence-based treatments for autistic adolescents, it is important to consider issues around the optimal delivery and access to interventions. For autistic individuals, there may be significant difficulties accessing face-to-face interventions delivered by clinicians with the specialist knowledge and skills required to deliver autism-specific interventions (Khan et al., 2019). Therefore, the development of interventions that can be delivered remotely is of particular interest for this population. In both non-autistic and neurodivergent populations direct comparisons between online and in person psychotherapy for depression have found similar outcomes in terms of efficacy for reducing depressive symptoms, as well as similar levels of drop-out (Giovanetti et al., 2022; Khan et al., 2019). There are few studies testing remote interventions in those with autism, but those that do suggest that they show efficacy, feasibility and acceptability (Adams et al., 2022), and remote interventions specifically using video conferencing has shown promise in autism populations (e.g. McCrae et al., 2021). However, despite these positive indications, there are several possible barriers. Some studies have highlighted that when the intervention is primarily in the form of guided self-help there may have been too little support for some individuals (Westerberg et al., 2021). This suggest that at least initially interventions should be developed with comparable clinician contact times, regardless of the mode of delivery.

Given the high promise of both BA and remote delivery as treatment options for autistic people this study aimed to work with clinicians and autistic adolescents to develop a BA-based intervention which was then piloted using remote delivery. This work was informed by that of Russell et al. (2020) but with adaptations made to the intervention materials and content to account for its use in adolescence and following co-design sessions Additionally, with the intention to develop a therapist-led intervention inclusive to those with complex mental health needs who may not benefit from a low intensity approach.

## Methods

This study uses a case series design to explore the feasibility and acceptability of delivering an online BA intervention with autistic adolescents displaying moderate-to-severe depression. Seven participants were recruited into the study (mean age = 14.86, range 13–17 years), with six completing the intervention (see Table 1 for inclusion/exclusion criteria). Participants were referred to the intervention from a Child and Adolescent Mental Health services with the primary presenting problem of depression in the context of a clinically confirmed autism diagnosis, with two individuals having a co-occurring presentation of social anxiety. The case series was preceded by a period of co-design with autistic adolescents (different from those enrolled into the study) and clinicians, with input from these groups being integrated into the design of the intervention. Ethical approval was granted for this study from Brent Research Ethics Committee (IRAS: 303173, REC reference: 22/LO/0368). This study used the TIDIER checklist (Hoffmann et al., 2014) to report the current intervention (Appendix A).

**Table 1.** Study Inclusion and Exclusion Criteria.

Inclusion criteria	Exclusion criteria
i) Aged between 13 and 19 years of age	i) Adolescents with co-occurring psychiatric conditions such as obsessive-compulsive disorder and post traumatic stress disorder
ii) A confirmed diagnosis of autism spectrum disorder	ii) Those already undergoing other psychotherapies (e.g. CBT)
iii) A diagnosis of depression, or moderate-severe symptoms of depression	iii) Those at high risk of harming themselves or others
iv) Participant under the care of a local child and adolescent mental health service	
v) Psychiatric medication likely to remain stable throughout the intervention	
vi) Participant has sufficient spoken English to access the intervention	
vii) Verbal/intellectual ability in the average range (a scaled IQ score of 80 and above)	

### Co-design with autistic youth and clinicians

An initial version of the intervention protocol was presented, along with some example resources taken from the ADEPT (Guided self-help for depression in adults with autism) study BA intervention for adults (Russell et al., 2020), to a group of autistic adolescents (*n = 4*), and clinicians (*n* = 4), to gain feedback and make adaptations to the intervention protocol. Adolescents and clinicians were recruited from neurodevelopmental mental health services in South London and had varying experiences of receiving or delivering psychological interventions, respectively. Key suggestions raised by the adolescent group integrated into the intervention approach included: (a) offering flexibility in session day and time to fit with other activities; (b) providing a written summary of session context between sessions as a reminder; (c) an extended initial period of rapport building and getting to know the young person; (d) for the therapist to use a ‘getting to know you sessions’ to support the young person with activity scheduling; (e) support with emotional literacy skills; (f) to take a graded approach to activity scheduling, and (g) to involve parents where possible to support delivery. Clinician input identified that whilst a default session length of 60 minutes was fine, flexibility in session length and order of intervention sessions is required. Common difficulties with problem solving in autism and with young people engaging in unhelpful behaviours which reinforce low mood were also highlighted. Following the co-design sessions three optional sessions were added to the intervention focusing on ‘other emotions’, problem solving, and unhelpful behaviours.

### Behavioural activation intervention for autistic adolescents

The BA intervention consisted of 12 one-to-one sessions, delivered remotely via videocall. Each was designed to last up to 1 hour, but with flexibility for shorter sessions depending on the individual and whether, for example, they were able to sustain their attention/engagement for the whole hour. All the interventions were delivered by a trainee Clinical Psychologist, a registered Clinical Psychologist, or a post-doctoral research psychologist under the supervision of a registered Clinical Psychologist. Parents were invited to be a part of the intervention, and input was tailored based on the preference of the participant and the overall need to support treatment progress. Across the intervention, the following content was included for all participants: (i) getting to know the participant and their interests; (ii) goalsetting (SMART goals); (iii) psychoeducation around depression and concepts of behavioural activation in the context of autism, (iv) mood and activity monitoring; (v) activity scheduling; (vi) reviewing and recapping therapy. The following topics were included as optional modules (i) emotion regulation skills; (ii) problem solving skills; (iii) identifying and overcoming unhelpful behaviours which may maintain depression. If the optional sessions were not used, then the session defaulted to activity monitoring and scheduling, or to recap on content not covered or clearly understood in previous sessions. Participants were asked to record their physical activity using Fitbit^TM^ devices to be reviewed alongside mood and activity diaries at the start of each session.

### Measures of acceptability and feasibility

Feasibility for this intervention was assessed using participant retention and drop-out rates. Acceptability was assessed by measuring satisfaction with the content and delivery of the intervention sessions, and completion of between session homework. This feedback was gathered using semi-structured interviews after session four, eight and twelve. Young people and parents contributed to acceptability feedback for this study using a predesigned interview schedule. Completion rates for both parent and self-report clinical outcome measures were used as a secondary measure of acceptability, and were completed before, midway, and after the intervention was complete.

### Clinical measures

The Social Communication Questionnaire (SCQ; Rutter et al., 2003) – lifetime form. The SCQ is a brief 40-item screening questionnaire for autism symptoms and behaviours, designed to be completed for primary caregivers of young people aged 4 and over. Diagnostic validity studies have suggested that a score greater or equal to 15 is indicative of effective screening for autism (Berument et al., 1999). The SCQ has also been shown to have good validity and efficacy in a UK population (Chandler et al., 2007). The SCQ was completed at baseline only to characterise the sample.

#### The beck depression inventory – second edition (BDI-II; Beck et al., 1996)

The BDI-II is a brief 21-item self-report measure, designed to screen and measure the nature of depressive symptomology. The BDI-II measures depressive symptoms over the previous 2 weeks. It has been shown to have high reliability, able to distinguish between depressed and non-depressed presentations, and improved validity compared to its previous version (Wang & Gorenstein, 2013) and has been used previously with autistic adolescents (Gotham et al., 2015).

#### The revised children’s anxiety and depression scale (RCADS) – self- and parent-report (Chorpita et al., 2000)

The RCADS consists of 47 questions, designed to be self-reported by people aged 8–18 years. It includes subscales measuring symptoms of depression, generalized anxiety, separation anxiety, obsessive compulsive disorder, social phobia, and panic disorder. The RCADS has been shown to be consistent with DSM criteria of depression and anxiety (Chorpita et al., 2000) and there is evidence to support its use with autistic young people (Sterling et al., 2015). The RCADS and RCADS-parent report were completed at baseline, midpoint and at the end of the intervention.

#### Remote monitoring of physical activity

For the duration of the intervention, participants were asked to wear a Fitbit^TM^ device to monitor number of steps and distance covered during the week between therapy sessions. Fitbit^TM^ devices were provided to participants, and they were allowed to keep these at the end of the study. This data was collected in the first 5 minutes of the sessions and therapists were able to use this data to have conversations about activity levels as a part of the intervention. Due to variable usage of the Fitbit^TM^ across participants, the data obtained will only be considered in terms of acceptability and not as a clinical outcome in this study.

### Statistical analysis

Measures of acceptability and feasibility are presented descriptively. The Reliable Change Index (RCI, Jacobson & Truax, 1992) was used to determine any reliable improvement for each key outcome measure at an individual level. Jacobson and Truax (1992) suggested that reliable change is calculated as the post-intervention scores minus the pre-intervention scores, divided by the standard error of the difference of these two scores. If the score is greater than 1.96 times the standard error difference of the two scores, then Jacobson and Truax (1992) suggested it is “unlikely that the post-test score is not reflecting real change”. The RCI score indicates the difference in post-test score needed on a measure to suggest this change. The formula used is shown below and was calculated using the Leeds reliable change calculator (Morley & Dowzer, 2014), consistent with previous studies using the RCI.

$${\text{SE}}_{\text{meas}}=\text{SD}\;*\;\sqrt{(1}-\text{reliability})$$

$$\text{RCI}=\left(posttest-pretest\right)\;/\;{\text{SE}}_{\text{meas}}$$

Measurement of deviations were obtained from the current sample pre-intervention, and the reliability data (Cronbach’s alpha) was obtained from the relevant papers: Ebesutani et al. (2015); Kösters et al. (2015); and Gotham et al. (2015). The RCI values for each measure are as follows: BDI-II = 17.59; RCADS Depression = 9.08; RCADS-P Depression = 7.26; RCADS Anxiety = 14.22; and RCADS-P Anxiety = 11.62. As the primary focus of this study is acceptability and feasibility, all statistical analysis should be interpreted as preliminary findings.

## Results

The sample included seven autistic adolescents (Male = 5; Female = 2) aged between 13 and 17 years, with a clinically confirmed autism diagnosis, who consented to receive the remotely delivered BA intervention. 43% of participants were White British, but there were a range of ethnicities within the sample (See Table 2 for descriptive statistics). The mean SCQ score at pre-intervention was above the clinical cut off (M = 28.29, SD = 4.35). All participants scored in the clinical range (T-score >65) on the parent reported RCADS for both depression and anxiety suggesting this group had clinically significant symptoms across both domains.

**Table 2.** Descriptive Statistics.

Demographic	*n* (%)
Age	
13–14	3 (43%)
15–16	3 (14%)
17–18	1 (14%)
Gender	
Male	5 (71%)
Female	2 (29%)
Ethnicity	
White british	3 (43%)
White european	1 (14%)
Black british	2 (29%)
British arabian	1 (14%)
Social communication measure baseline (*n* = 7)	Mean (range)
Social communication questionnaire (0–39)	28.29 (20–32)
Depression measures baseline (*n* = 6)	
BDI-II (0–63)	24.66 (9–42)
RCADS-C depression (0–30)	13.83 (6–23)
RCADS-P depression (0–30)	18.33 (11–28)
Depression measures post-intervention (*n* = 6)	
BDI-II (possible range: 0–63)	21.83 (8–44)
RCADS-C depression (0–30)	12.00 (4–23)
RCADS-P depression (0–30)	17.66 (9–26)
Anxiety measures baseline (*n* = 6)	Mean (range)
RCADS-C anxiety (0–30)	41.83 (20–58)
RCADS-P anxiety (0–30)	61.83 (36–87)
Anxiety measures post-intervention (*n* = 6)	
RCADS-C anxiety (0–30)	41.50 (11–67)
RCADS-P anxiety (0–30)	48.66 (20–74)

### Measures of feasibility and acceptability

#### Feasibility

Fifteen cases (53% male, 47% female) were screened for the intervention, who were between the ages 13–17.4 years, having been identified by child mental health services based on the inclusion criteria below. Seven screened participants where not enrolled in the study for a number of reasons, including not meeting study criteria (e.g. being below the threshold for IQ level, inappropriate for this intervention due to co-occurring conditions), or not consenting to be contacted. Overall, 73% of referrals that were screened, were contacted. Of the 11 who were screened and approached (64% male, 36% female), 4 declined to participate. This was due to accepting another intervention elsewhere (e.g. from school intervention or local mental health provider), or not wanting to take part in the intervention. Of those who were screened and contacted, seven cases (64%) took part in the intervention (5 male, 2 female). One participant dropped out of the intervention (making the overall rate of intervention uptake 55%), with complete outcome measures from the remaining six being high with 100% of parent measures being completed pre-intervention, mid-intervention, and post-intervention. 100% of child measures were completed pre-intervention, 83% completed mid-intervention, and 100% completed post-intervention.

#### Acceptability

Participant were considered to be retained in the study if they completed both pre- and post-intervention measures. Overall, six out of seven participants were retained to post-intervention measures, and of those 5/6 of participants completed eleven or more sessions. The average number of sessions attended was 9 and a median of 11 sessions (range = 8–12). We report on the six participants who completed the full intervention only.

Five of participants completed at least one between session homework task, and out of the six activities that could be set during the intervention, five of the participants completed at least five activities. However, mood diaries were not completed between sessions by any participant, and instead were completed in sessions.

Regarding in-session treatment fidelity, all participants completed the “getting to know you” worksheet, 5/6 developed SMART goals, and all of participants completed depression psychoeducation, mood monitoring, activity scheduling, and the therapy review. 3/6 of participants completed the optional flexible session on other emotions (emotional regulation strategies), One participant completed the optional problem-solving session, and one completed the optional unhelpful behaviours session. Participants reported Fitbit^TM^ data from the previous week during on average 8 sessions (range 5–12). Half of the participants collected Fitbit^TM^ data for eight weeks or more, and 33% of participants collected data for 11 weeks or more.

Based on post-intervention interviews, all participants who completed the intervention would recommend it to other autistic adolescents and found it to be a positive experience. No participants reported the intervention to be a negative experience. All participants reported that the online delivery was an acceptable format of receiving psychological therapy, and 4/6 reported they preferred videocalls to in person sessions. All participants were happy with the weekly format of the intervention, and 5/6 were happy with the hour length, although flexibility in session time was reportedly important. One reported that a shorter session was necessary. From overall parent feedback, all parents recommended this intervention to other autistic adolescents, and found this intervention to be a positive experience for their child. No parents reported the intervention to be a negative experience.

#### Clinical outcomes

The mean pre- and post-intervention depression scores for individual participants can be seen in Table 2 and change scores for each individual are in Table 3. One of the six participants showed a reliable change (see the Statistical Analysis section for a definition of reliable change) in depression scores and four out of six showed reliable change in anxiety symptoms based on parent report, with all participants scores reducing from pre-to post-intervention. Four participants showed a reliable change (i.e. reduction) in scores on at least one clinical outcome (parent- or self-reported).

**Table 3.** Individual Change Scores Across Depression and Anxiety Outcome Measures Scores.

	Change in BDI-II	Change in RCADS depression	Change in RCADS-P depression	Change in RCADS anxiety	Change in RCADS-P anxiety
Case 1	+2	−2	−6	+5	−11†
Case 2	+2	+2	−2	+9	−13*
Case 3	−1	−4	−0	−9	−18*
Case 4	−4	0	+7	−9	−3
Case 5	N/A	N/A	N/A	N/A	N/A
Case 6	−21*	−10*	−5	−9	−18*
Case 7	+5	+3	+2	+13	−16*

BDI-II = Beck Depression Inventory; RCADS = Revised Children’s Anxiety and Depression Scale; RCADS-P = The Revised Child Anxiety and Depression Scale – Parent Version; * = reliable change on that outcome measure; † = borderline reliable change.

## Discussion

To our knowledge this is the first study to present data on a co-designed BA based intervention designed for use with autistic adolescents. Our pilot case series demonstrates that the intervention and its delivery in a remote format was acceptable to autistic adolescents and their parents. All but one of the young people taking part in the intervention was retained until endpoint, with everyone recommending it for future use. The low dropout rate on agreement to participate, and good outcome measure completion in this pilot case series indicates that the intervention has promise and warrants further investigation in respect of effectiveness.

A retention rate of at least 80% is normally recommended as an indicator of acceptability for an intervention study (Fewtrell et al., 2008), suggesting that our rate of 86% is indicative of an acceptable intervention. We also found that most participants attended all but one of the 12 sessions offered. One area in which participants struggled was the completion of between session mood and activity monitoring using the paper diaries provided. This may be related to difficulties around organisation and planning (Demetriou et al., 2018) but also possibly due to these being completed using ‘paper-and-pencil’. A future study may benefit from integrating digital materials for mood monitoring and activity scheduling, including the incorporation of between session reminders.

A major emphasis of this work has been on the early inclusion and co-design of session content, adaptations and materials with autistic young people and clinicians with the aim to boost acceptability of the intervention and in the longer term enhance the efficacy and effective implementation into clinical services. The co-design process of the intervention influenced the design of the intervention manual and session content by placing an emphasis on flexibility, both in terms of session time/length but also the pacing of session content. This meant that we built flexibility into the session order, making some sessions optional, and allowing time to repeat or extend session content to meet the needs of the individual. This was balanced with ensuring all the key intervention content was covered across the 12 sessions. Other feedback was in line with more established recommendations around autism specific adaptations, such as the inclusion of emotional literacy and regulation work, and an extended period of rapport building (Chew et al., 2022). An important feedback point was that parent involvement should be an option, even in the older adolescents who may need support with accessing session content and organisation, as well as some support generalising session content into real world settings.

Interestingly, the inclusion of a graded approach to activity scheduling (as recommended by autistic adolescents) overlaps with the theoretical underpinnings of anxiety-based interventions (Chorpita & Daleiden., 2009), whereby in the initial stages the scheduled activities are easy to achieve and possibly extensions to those already being participated in. New activities then build slowly on these, thereby reducing barriers related to high anxiety. It is important to note that autistic adolescents are at high risk of experiencing clinical levels of anxiety alongside depression (Hollocks et al., 2022), and this was indeed the case in this clinically representative group, with all participants scoring in the clinical range for anxiety. This adaptation and through increasing exposure to activities in a supportive manner may explain why we found out greatest reduction in symptoms on the anxiety measures, rather than in depression. It is also important to note the inclusion of sessions on emotional literacy and regulation are likely targeting transdiagnostic mechanisms (Weiss, 2014) with improvement in these skills likely impacting both sets of symptoms. Previous work in non-autistic samples has suggested that the management of anxiety is an important component of preventing and treating depression (Flannery-Schroeder., 2006). It is possible that in this complex population a longer intervention length (i.e., greater number of sessions) is required for the effects of reducing anxiety to translate into improved mood.

This study has several strengths, including the emphasis on co-design and recruitment and delivery of the intervention in a ‘real-life’ clinical sample of adolescents with complex mental health needs. However, it is important to put this in the context of some limitations. Whilst most young people found the sessions positive, for some it was hard to manage the remote completion of between session homework and activity monitoring. Our future research will focus on incorporating this feedback to enhance the intervention further before conducting further evaluation. Similarly, the use of the Fitbit to record physiological activity was not well received and was used inconsistently. Therapist feedback suggested that whilst it was useful when recorded to support discussions about activity levels, it could be considered a poor outcome measure as activity levels as measured by the device (number of steps etc.) were affected by issues external to the intervention, such as illness or not remembering to wear the devise. More broadly, this study would have benefited from a more detailed recording of intervention adherence. This could have included reporting of which specific components of the intervention were not completed and reasons given for this. This will be an important addition to future studies.

It is also important to note that, consistent with most studies investigating psychological treatment for autistic people (Wang et al., 2021), changes in outcome measures were only identified based on parent report. Whilst this was not the focus of the current study it will be important to consider how best to detect change in autistic adolescents based on self-report. Finally, whilst overall the experience of the study participants was positive, an overall uptake rate of 55% means that perhaps only a subsample of autistic individuals who were very motivated to receive an intervention took part.

Overall, we can conclude that this BA based intervention for depression is both acceptable and feasible for use with autistic adolescents, and that there are preliminary indicators of efficacy which should be investigated in a larger scale study with a randomised design. Where possible, incorporating further improvements based on the feedback of autistic adolescents and their parents and with a greater focus on establishing treatment efficacy, is warranted.

## Appendix

### Appendix A: TIDIER Checklist (Hoffman et al., 2014) Completed for the Study

The TIDieR (Template for Intervention Description and Replication) Checklist*:

Information to include when describing an intervention and the location of the information

**Table:**

Item number	Item	Where located **		
		Primary paper (page or appendix number)	Other^†^ (details)	
	**BRIEF NAME**		
**1**	Provide the name or a phrase that describes the intervention	4	______________
	**WHY**		
**2**	Describe any rationale, theory, or goal of the elements essential to the intervention	2, 3	_____________
	**WHAT**		
**3**	Materials: Describe any physical or informational materials used in the intervention, including those provided to participants or used in intervention delivery or in training of intervention providers. Provide information on where the materials can be accessed (e.g. online appendix, URL)	5, 6	_____________
**4**	Procedures: Describe each of the procedures, activities, and/or processes used in the intervention, including any enabling or support activities	5,6,7	_____________
	**WHO PROVIDED**		
**5**	For each category of intervention provider (e.g. psychologist, nursing assistant), describe their expertise, background and any specific training given	5	_____________
	**HOW**		
**6**	Describe the modes of delivery (e.g. face-to-face or by some other mechanism, such as internet or telephone) of the intervention and whether it was provided individually or in a group	5	_____________
	**WHERE**		
**7**	Describe the type(s) of location(s) where the intervention occurred, including any necessary infrastructure or relevant features	5	_____________
	**WHEN and HOW MUCH**		
**8**	Describe the number of times the intervention was delivered and over what period of time including the number of sessions, their schedule, and their duration, intensity or dose	5	_____________
	**TAILORING**		
**9**	If the intervention was planned to be personalised, titrated or adapted, then describe what, why, when, and how	4,5,6	_____________
	**MODIFICATIONS**		
**10.** ^**ǂ**^	If the intervention was modified during the course of the study, describe the changes (what, why, when, and how)	N/A	_____________
	**HOW WELL**		
**11**	Planned: If intervention adherence or fidelity was assessed, describe how and by whom, and if any strategies were used to maintain or improve fidelity, describe them	6	_____________
**12.** ^**ǂ**^	Actual: If intervention adherence or fidelity was assessed, describe the extent to which the intervention was delivered as planned	10	_____________
